# Endocrine advantages of PD-1/PD-L1 therapy: Comparative analysis of FAERS-JADER

**DOI:** 10.1371/journal.pone.0340794

**Published:** 2026-01-09

**Authors:** Yuxuan Gao, Shiyao Jiang, Yu Cui, Yumeng Wang, Lili Yu

**Affiliations:** 1 Department of Public Health, Shandong Second Medical University, Weifang, Shandong, China; 2 Department of Medicine Information, The 960th Hospital of the PLA Joint Logistics Support Force, Jinan, Shandong, China; University of Arkansas for Medical Sciences, UNITED STATES OF AMERICA

## Abstract

With the extensive clinical application of immune checkpoint inhibitors (ICIs), immune-related adverse events (irAEs) associated with these agents have increasingly garnered significant attention. Unlike other irAEs, endocrine irAEs are mostly irreversible, with variable and nonspecific symptoms, which poses challenges for clinicians in diagnosis. As a result, this study leveraged the U.S. Food and Drug Administration Adverse Event Reporting System (FAERS) and the Japanese Adverse Drug Event Report (JADER) pharmacovigilance databases to conduct an in-depth investigation into adverse events induced by PD-1/PD-L1 inhibitors, with a focus on irAEs induced by PD-1/PD-L1 inhibitors. This study pioneers the systematic cross-database validation of endocrine irAEs induced by PD-1/PD-L1 inhibitors. The integration of data from the JADER offers unique safety insights for Asian populations, bolsters global pharmacovigilance efforts, and uncovers regional variations in irAEs reporting. Notably, this study revealed a higher prevalence of endocrine irAEs among men aged over 50 years receiving PD-1/PD-L1 inhibitors. Both PD-1 and PD-L1 inhibitors are strongly associated with thyroid dysfunction, adrenal insufficiency, and pituitary inflammation. Additionally, it identifies several previously undocumented endocrine irAEs. This result unearthed safety signals hitherto unreported in drug inserts, underscoring the imperative for updating the safety labeling of PD-1/PD-L1 inhibitors with respect to endocrine irAEs. The emergence of off-label uses further underscores the need for additional clinical trials to assess their efficacy and safety.

## Introduction

Immune checkpoint inhibitors (ICIs) have revolutionized cancer therapy. By precisely targeting and inhibiting immune checkpoint molecules, they enhance the body’s immune response against tumor cells at various stages [1]. As one of the most successful strategies in cancer treatment to date, ICIs have been extensively applied in treating diverse malignant tumors, such as non-small cell lung cancer, melanoma, and colorectal cancer [2]. On the basis of different targets, ICIs can be classified into several categories, including anti-cytotoxic T-lymphocyte-associated antigen 4 (CTLA-4), anti-programmed cell death receptor-1 (PD-1), anti-programmed death-ligand-1 (PD-L1), and lymphocyte activation gene 3 (LAG-3) [3]. Among these, PD-1/PD-L1 inhibitors are the most prevalent. Since 2014, the U.S. Food and Drug Administration (FDA) has approved multiple PD-1/PD-L1 inhibitors for marketing, namely, nivolumab, pembrolizumab, tislelizumab, cemiplimab, dostarlimab, toripalimab, atezolizumab, durvalumab, and avelumab.

Despite the remarkable therapeutic efficacy of PD-1/PD-L1 inhibitors in managing malignant tumors, their mechanism of action—by altering the body’s immune tolerance—inevitably elevates the risk of autoantigen-mediated immune responses. This mechanism has led to frequent reports of immune-related adverse events (irAEs) in the majority of treated patients [4]. The most common irAEs are skin-related adverse reactions, gastrointestinal disorders, and endocrine disorders [5]. Among endocrine irAEs, the thyroid, pituitary, and adrenal glands are the most commonly affected organs in that specific order [6]. Notably, endocrine irAEs differ from other irAEs in their often irreversible nature, frequently necessitating lifelong hormone replacement therapy [7]. Owing to this challenge, the symptoms of endocrine dysfunction are highly variable and nonspecific, posing diagnostic difficulties for clinicians. This complexity is further exacerbated by the widespread use of combined corticosteroids [8].

Unlike previous studies, this investigation distinctively leverages the U.S. Food and Drug Administration Adverse Event Reporting System (FAERS) and the Japanese Adverse Drug Event Report (JADER) pharmacovigilance databases to conduct a comprehensive safety assessment of PD-1/PD-L1 inhibitor-induced endocrine irAEs [9]. In particular, the FAERS collates global case reports with a primary focus on the U.S. [10], whereas the JADER exclusively includes adverse reaction data from Japan [11]. Moreover, while recent research has focused predominantly on broad-spectrum irAEs associated with ICIs, a conspicuous gap remains in target-specific investigations of individual irAEs types [12].

Thus, this study aims to employ cross-database comparative validation to explore the phenotypic expression of endocrine irAEs across different PD-1/PD-L1 inhibitors and populations. By systematically analyzing the FAERS and JADER datasets, this research seeks to 1) identify risk profiles for immune-related adverse endocrine reactions; 2) uncover potential drug‒toxicity correlations; and 3) provide clinicians with evidence-based insights for proactive risk mitigation [13].

## Method

### Study design and data sources

This study analyzed adverse endocrine reactions associated with PD-1/PD-L1 inhibitors via data from the FAERS database (Q1 2005 to Q3 2024). As a self-reported repository, the FAERS collects adverse drug reactions from pharmaceutical companies, healthcare professionals, and consumers, serving as a public resource to support the FDA’s monitoring of approved drugs and biologics in the U.S. [14]. Specifically, the FAERS comprises seven tables encompassing patient demographics, drug information, adverse reaction details, report sources, treatment timelines, indications/diagnoses, and case deletions.

The analytic environment for this study consisted of R software (version 4.3.3) with the dplyr and tidyr packages for data management. The initial FAERS data retrieval and consolidation were performed using OpenVigil 2.1 [15] (https://openvigil.sourceforge.net/). Following FDA guidelines, the resulting data files from each quarter were then merged and subjected to a standardized data cleaning and harmonization procedure within the R environment. The specific workflow/procedure is as follows:

(1) Deduplication procedure: Case reports were deduplicated based on the FDA-recommended primary case identifier (primaryid) in combination with key demographic and event variables, including caseid, age, sex, event date (event_dt), and drug role code (role_cod). When duplicate records were identified, the record with the most recent FDA receipt date (fda_dt) was retained for analysis to ensure the most up-to-date information was used.
(2) Inclusion criteria: For the primary analysis, reports were included only where the PD-1/PD-L1 inhibitors of interest was listed as a ‘Primary Suspect (PS)’ drug. This criterion was chosen to strengthen the causal inference between the drug and the reported adverse event. Reports where the drug was listed as a ‘Secondary Suspect’ or ‘Concomitant’ were excluded from the primary analysis to minimize confounding.
(3) Handling of missing data: Cases with completely missing demographic information (both age and sex) were excluded from the analysis. For cases with partial missing data, the available information was utilized without imputation. Specifically, reports with missing age or sex were retained and included in analyses where the respective variable was not required.

In this study, adverse events (AEs) were coded according to the Preferred Terms (PTs) of the Medical Dictionary for Regulatory Activities (MedDRA version 25.0) to ensure standardized and precise description of medical conditions [16]. PD-1/PD-L1 inhibitors were searched via a list of generic names/ brand names, “pembrolizumab/Keytruda”, “nivolumab/Opdivo”, “cemiplimab/Libtayo”, “atezolizumab/Tecentriq”, “durvalumab/Imfinzi”, “avelumab/Bavencio”, —designated primary suspects. Endocrine-related adverse reactions were then screened, yielding 8,277 extracted reports.

In the JADER database, identical search terms as those used in FAERS were applied, designating them as primary suspects. The retrieved data were subsequently cleaned and sorted via R 4.3.3 software, following the FDA-recommended deduplication strategy. After the data were standardized with the MedDRA-v25.0 lexicon, an analysis was conducted on adverse endocrine effects associated with PD-1/PD-L1 inhibitors from January 2004 to January 2025.

In this study, we excluded three monoclonal antibodies among the top nine FDA-approved PD-1/PD-L1 inhibitors: tislelizumab, toripalimab and dostarlimab. The primary reason is that these drugs entered the market relatively late after FDA approval—all after 2019, with two inhibitors launched after 2021, resulting in significantly insufficient adverse event reports in the FAERS and JADER databases. Preliminary searches indicate that, as of the data cutoff date, the number of individual positive irAEs for these drugs was below 107. Such limited data volumes result in low statistical power when conducting disproportionate analysis, making it highly susceptible to unstable and unreliable signals. To avoid these potential biases and ensure the overall robustness and comparability of our cohort analysis results, we decided to focus our analysis on the six PD-1/PD-L1 inhibitors with longer market presence and more comprehensive reporting. Among these, the six inhibitors include three PD-1 inhibitors: nivolumab, pembrolizumab, and cemiplimab; and three PD-L1 inhibitors: atezolizumab, durvalumab, and avelumab.

### Statistical analysis

A four-compartment table based on the proportional imbalance method was employed to screen potential irAEs signals (Table 1). In particular, the reporting odds ratio (ROR) and proportional reporting ratio (PRR) were utilized, with calculations of the ROR, 95% confidence interval (95% CI), PRR, and chi-square (x²) performed to identify adverse reactions. In addition to the frequentist methods (ROR and PRR), the Empirical Bayes Geometric Mean (EBGM) was employed as a Bayesian data mining approach. This method applies statistical shrinkage to account for the variability in reporting frequencies, particularly for drug-event combinations with few cases, thereby providing more stable estimates and reducing the likelihood of false-positive signals. For specific formulas, see Table 2.

**Table 1:** Proportional imbalance method four-cell table.

Project	Targeted Adverse Event Reporting	Other Adverse Event Reports	Total
Target Drug	a	b	a + b
Other Drugs	c	d	c + d
Total	a + c	b + d	n = a + b + c + d

^a^Number of reports of suspected adverse events for suspected drugs.

^b^Number of reports of nonsuspected adverse events for suspected drugs.

^c^Number of reports of suspicious adverse events for nonsuspicious drugs.

^d^Number of reports of nonsuspicious adverse events for nonsuspicious drugs.

**Table 2:** Algorithm for drug safety signal detection.

Algorithms	Formulas	Threshold
ROR	$\text{ROR}=\frac{\text{a}/\text{c}}{\text{b}/\text{d}}=\frac{\text{ad}}{\text{bc}}$	95% CI > 1.00, a ≥ 3
	$\text{9}\text{5}\%CI=e^{ln(ROR)\pm \text{1}.\text{9}\text{6}\sqrt{\frac{\text{1}}{a}+\frac{\text{1}}{b}+\frac{\text{1}}{c}+\frac{\text{1}}{d}}}$	
PRR	$\text{PRR}=\frac{\text{a}(\text{c}+\text{d})}{\text{c}(\text{a}+\text{b})}$	PRR ≥ 2, x^2^ ≥ 4, a ≥ 3
	${\text{x}}^{\text{2}}=\frac{\left(\text{ad}-\text{bc}{)}^{\text{2}}(\text{a}+\text{b}+\text{c}+\text{d}\right)}{\left(\text{a}+\text{b})(\text{c}+\text{d})(\text{a}+\text{c})(\text{b}+\text{d}\right)}$	
EBGM	$\text{O}/\text{E}=\frac{\text{a}}{\text{E}}$	EB05 ≥ 2
	$\text{E}=\frac{\left(\text{a}+\text{b})(\text{a}+\text{c}\right)}{\text{a}+\text{b}+\text{c}+\text{d}}$	
	$\text{EBGM}=\frac{\text{a}+\text{0}.\text{5}}{\text{E}+\text{0}.\text{5}}$	

For the frequency-based methods, a signal was considered positive if the lower limit of the 95% CI of ROR exceeded 1 and the number of reports containing both the suspect drug and adverse event (a) was ≥ 3. For PRR, a signal was deemed positive if PRR ≥ 2, x^2^ ≥ 4, and a ≥ 3. For EBGM, a signal was considered positive if the lower limit of the 95% CI was greater than or equal to 2. In this study, only signals meeting the thresholds of all four algorithms (ROR, PRR, x², and EBGM) simultaneously were confirmed as positive and included in the final analysis.

To assess the robustness of the identified signals, we conducted the following sensitivity analyses:

(1) Minimum Case Threshold: We required a minimum of 3 reports for any drug-event combination to filter out unstable signals arising from very small case numbers.
(2) Cross-Database Validation: A signal was considered highly robust if it was statistically significant in both the FAERS and JADER databases.
(3) Alternative Outcome Definition: For signals of interest (e.g., SIADH), we tested their stability by expanding the event definition to include broader MedDRA terms.

## Results

### Number of reported cases of endocrine irAEs by country

**Fig 1:**
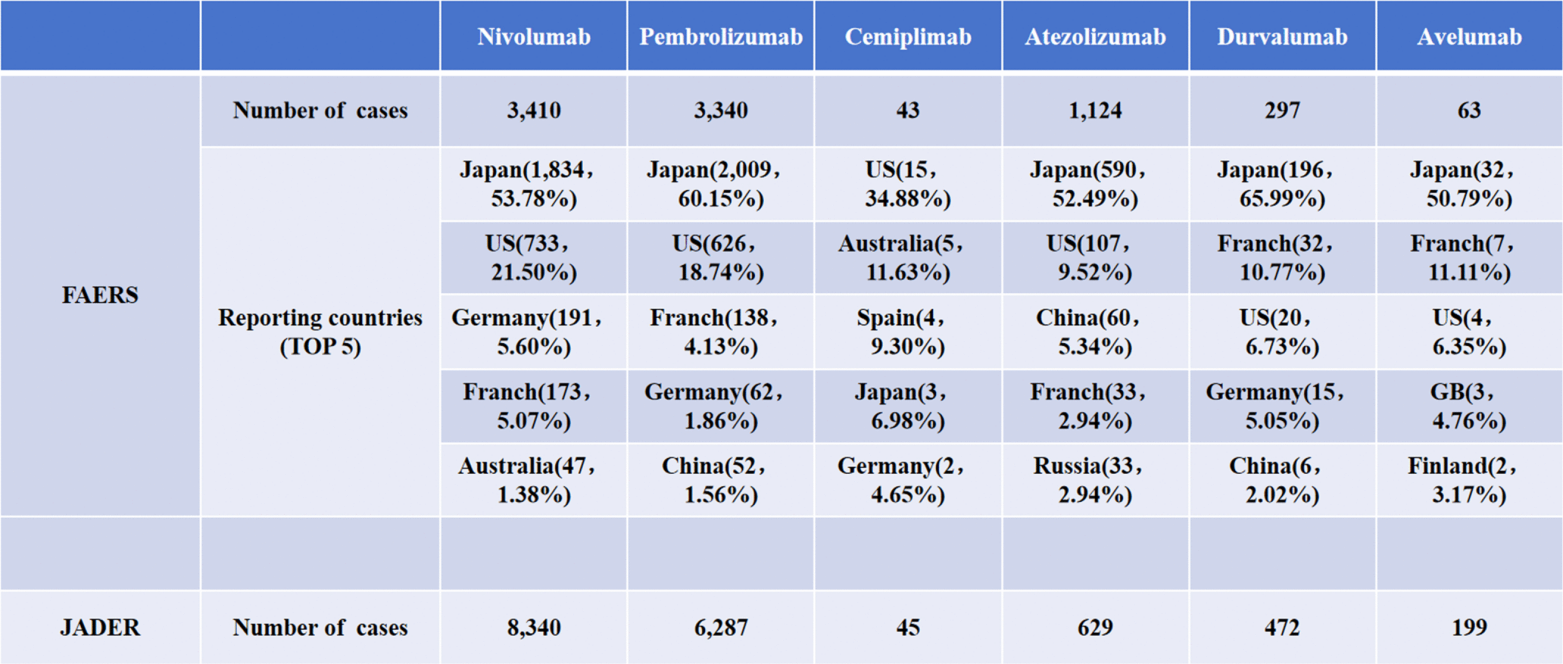
Number of reported cases of endocrine irAEs by country.

As of Q3 2024, the FAERS had screened a total of 8,277 endocrine irAEs, with case distributions as follows: nivolumab (3,410), pembrolizumab (3,340), cemiplimab (43), atezolizumab (1,124), durvalumab (297), and avelumab (63). Specifically, with the exception of cemiplimab, Japan submitted the highest number of reports for all other inhibitors. This underscores the significant contribution of Japanese data to the FAERS global pharmacovigilance system for these ICIs. In contrast, the JADER database recorded 15,972 cases, including 8,340 for nivolumab, 6,287 for pembrolizumab, 45 for cemiplimab, 629 for atezolizumab, 427 for durvalumab, and 199 for avelumab. The detailed data are presented in Fig 1.

Based on the reporting patterns of each drug, the higher reporting volumes for nivolumab and pembrolizumab in JADER compared to FAERS indicate their extensive use and monitoring in Japan. In contrast, cemiplimab had the lowest report counts in both databases, suggesting more limited clinical adoption. Notably, all three PD-L1 inhibitors had a similar or significantly higher number of reports in JADER than in FAERS.

### Correlations between endocrine irAEs and drugs

**Fig 2:**
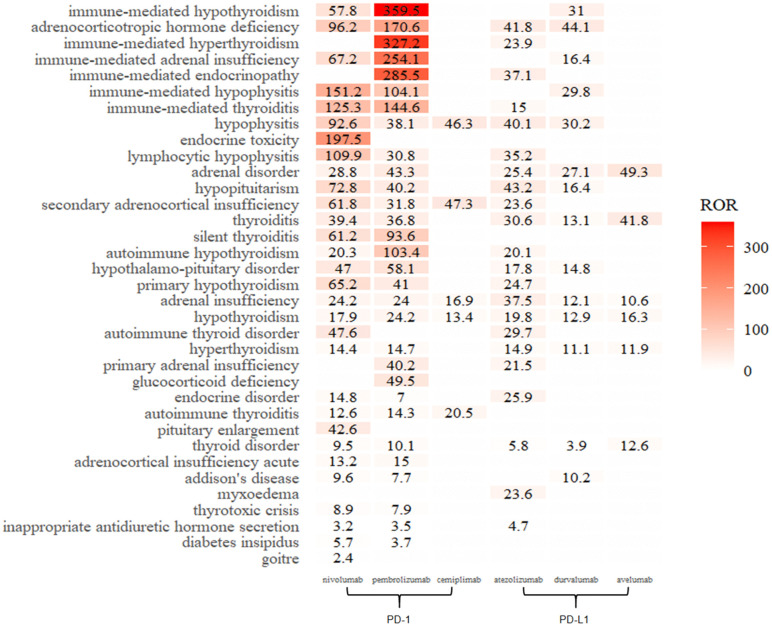
Correlations between endocrine irAEs and drugs—FAERS.

Fig 2 illustrates the correlation between endocrine irAEs and inhibitors in the FAERS. In particular, the top five irAEs associated with nivolumab were endocrine toxicity (ROR = 197.5), immune-mediated hypopituitarism (151.2), immune-mediated thyroiditis (125.3), adrenocorticotropic hormone deficiency (96.2), and hypopituitarism (92.6). Pembrolizumab was strongly correlated with immune-mediated hypothyroidism (ROR = 395.5), immune-mediated hyperthyroidism (327.2), immune-mediated endocrine disease (285.5), immune-mediated adrenal insufficiency (254.1), and adrenocorticotropic hormone deficiency (170.6). For cemiplimab, the top three correlations were secondary adrenocortical insufficiency (ROR = 47.3), inferior pituitary inflammation (46.3), and autoimmune thyroiditis (20.5). Atezolizumab was most strongly associated with hypopituitarism (ROR = 43.2), adrenocorticotropic hormone deficiency (41.8), hypopituitarism (40.1), and adrenal insufficiency (37.5). Similarly, the top five irAEs associated with durvalumab include adrenocorticotropic hormone deficiency (ROR = 44.1), immune-mediated hypothyroidism (31), hypopituitarism (30.2), immune-mediated hypopituitarism (29.8), and adrenal disease (27.1). Avelumab showed the strongest correlations with adrenal disease (ROR = 49.3), goiter (41.8), and thyroid disease (12.6).

Specifically, this study identified endocrine irAEs not documented in drug specifications compared with the corresponding inhibitor labels:

- Nivolumab: syndrome of inappropriate antidiuretic hormone secretion (SIADH) (ROR = 3.2), uremic avalanches (5.7);
- Pembrolizumab: hypopituitarism (ROR = 40.2), SIADH (3.5), urolithiasis (3.7);
- Atezolizumab: thyroiditis (ROR = 30.6), SIADH (4.7), and myxedema (23.6).

**Fig 3:**
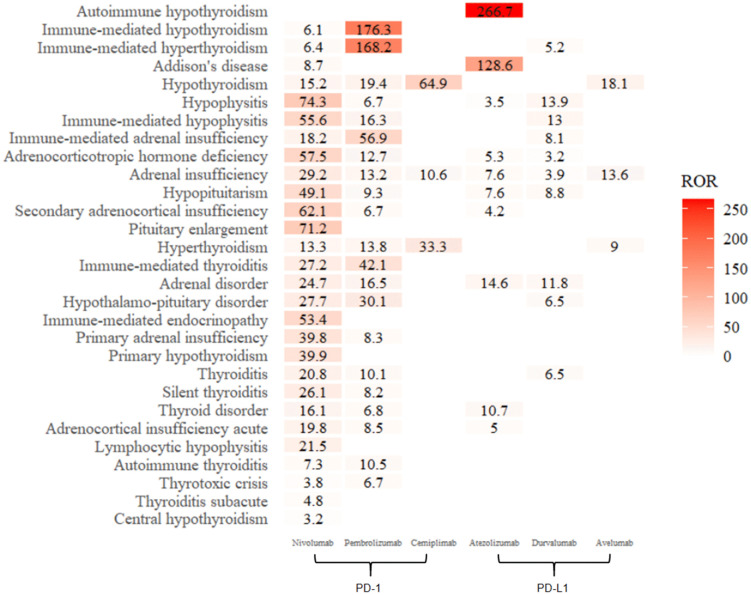
Correlations between endocrine irAEs and drugs—JADER.

Fig 3 depicts the correlation between endocrine irAEs and inhibitors in JADER. For nivolumab, the top five correlated irAEs were hypopituitarism (ROR = 74.3), an enlarged pituitary gland (71.2), secondary adrenocortical insufficiency (62.1), adrenocorticotropic hormone deficiency (57.5), and immune-mediated hypopituitarism (55.6). Pembrolizumab showed the strongest associations with immune-mediated hypothyroidism (ROR = 176.3), immune-mediated hyperthyroidism (168.2), immune-mediated adrenal insufficiency (56.9), immune-mediated thyroiditis (42.1), and hypothalamo-pituitary disease (30.1).

The three correlations with cemiplimab were hypothyroidism (ROR = 64.9), hyperthyroidism (33.3), and adrenal insufficiency (10.6). Atezolizumab demonstrated the greatest associations with autoimmune hypothyroidism (ROR = 266.7), Addison’s disease (128.6), adrenal disease (14.6), and thyroid disease (10.7). Durvalumab was most strongly associated with pituitary gland inflammation (ROR = 13.9), immune-mediated pituitary inflammation (13), adrenal gland disease (11.8), hypopituitarism (8.8), and immune-mediated adrenal insufficiency (8.1). The top five irAEs associated with avelumab include hypothyroidism (ROR = 18.1), adrenal insufficiency (13.6), and hyperthyroidism (9).

Specifically, this study identified endocrine irAEs not documented in drug specifications compared with the corresponding inhibitor labels:

- Pembrolizumab: hypopituitarism (ROR = 9.3);
- Atezolizumab: hypopituitarism (ROR = 7.6).

Cross-database comparisons revealed notable discrepancies in endocrine irAEs correlations. In FAERS, nivolumab demonstrated a strong association with immune-mediated thyroiditis, whereas JADER recorded a significantly lower ROR of 27.2 for the same irAEs. Conversely, FAERS showed a high correlation of cemiplimab with secondary adrenocortical insufficiency (ROR = 47.3), which was absent in JADER; in contrast, JADER revealed a prominent association of cemiplimab with hypothyroidism (ROR = 64.9) not observed in FAERS.

Atezolizumab exhibited marked database-specific differences: FAERS highlighted a strong pituitary disease correlation, whereas JADER showed an exceptionally high ROR of 266.7 for autoimmune hypothyroidism. The association of durvalumab with adrenocorticotropic hormone deficiency also varied significantly, with FAERS reporting ROR = 44.1 versus JADER’s ROR = 3.2. In concision, a cross-database comparison revealed both consistent and unique endocrine irAE signals (Table 3).

**Table 3:** Cross-database comparison of endocrine irAE signals.

Endocrine irAEs (MedDRA PT)	Drug Class	Drug(s) with Strong Signal	FAERS ROR	JADER ROR	Concordance
Hypophysitis	PD-1	Nivolumab	92.6	74.3	Yes
ACTH Deficiency	PD-1/PD-L1	Nivolumab, Pembrolizumab Durvalumab	96.2 (Nivolumab)	57.5 (Nivolumab)	Yes
Immune-mediated Hypophysitis	PD-1	Nivolumab	151.2	55.6	Yes
Immune-mediated Adrenal Insufficiency	PD-1	Pembrolizumab	254.1	56.9	Yes
Secondary Adrenocortical Insufficiency	PD-1	Cemiplimab	47.3	62.1 (Nivolumab)	Yes
Hypopituitarism	PD-L1	Atezolizumab	43.2	7.6	Yes
Immune-mediated Hypothyroidism	PD-1	Pembrolizumab	395.5	176.3	Yes
Immune-mediated Hyperthyroidism	PD-1	Pembrolizumab	327.2	168.2	Yes
Autoimmune Hypothyroidism	PD-L1	Atezolizumab	20.3	266.7	Yes
SIADH	PD-1/PD-L1	Nivolumab, Pembrolizumab, Atezolizumab	3.2-4.7	N/S	FAERS Only
Diabetes Insipidus	PD-1	Nivolumab, Pembrolizumab	5.7 (Nivolumab)	N/S	FAERS Only
Myxedema	PD-L1	Atezolizumab	23.6	N/S	FAERS Only
Pituitary Enlargement	PD-1	Nivolumab	N/S	71.2	JADER Only
Addison’s Disease	PD-L1	Atezolizumab	N/S	128.6	JADER Only

The detailed ROR calculation table (2 × 2 contingency table) for the key irAEs can be found in S1 Table.

### Indications for PD-1/PD-L1 inhibitors

Because of the low number of irAEs associated with cemiplimab, durvalumab, and avelumab, only nivolumab, pembrolizumab, and atezolizumab were considered indications for PD-1/PD-L1 inhibitors.

**Fig 4:**
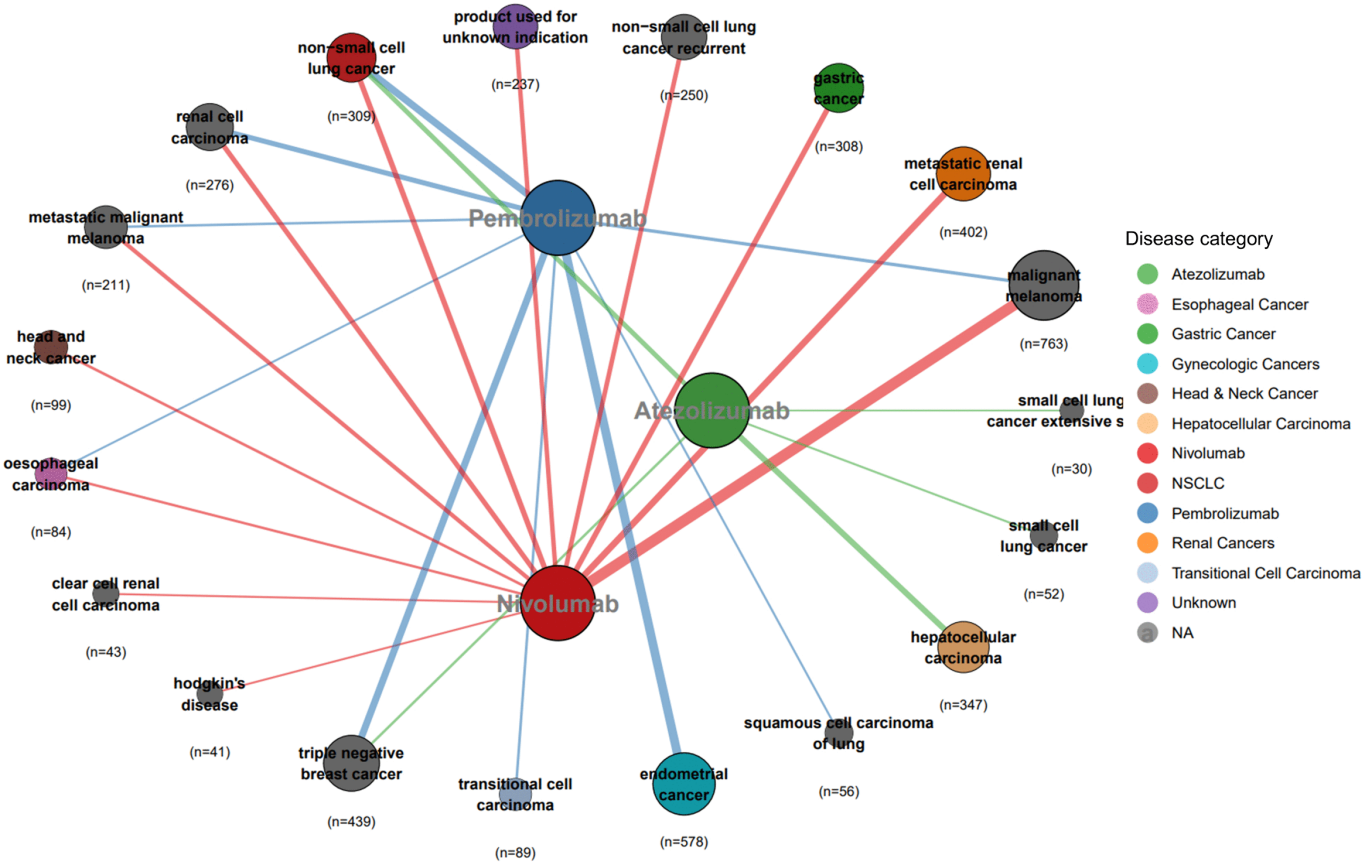
PD-1/PD-L1 Inhibitors Indications – FAERS (n = 4,614).

As shown in Fig 4, the FAERS data indicate that nivolumab is indicated for malignant melanoma, metastatic renal cell carcinoma, non-small cell lung cancer, gastric cancer, renal cell carcinoma, and head and neck cancer, among other cancers. Pembrolizumab is primarily used for treating endometrial cancer, triple-negative breast cancer, non-small cell lung cancer, and renal cell carcinoma, whereas atezolizumab is indicated for treating hepatocellular carcinoma, non-small cell lung cancer, and small cell lung cancer.

**Fig 5:**
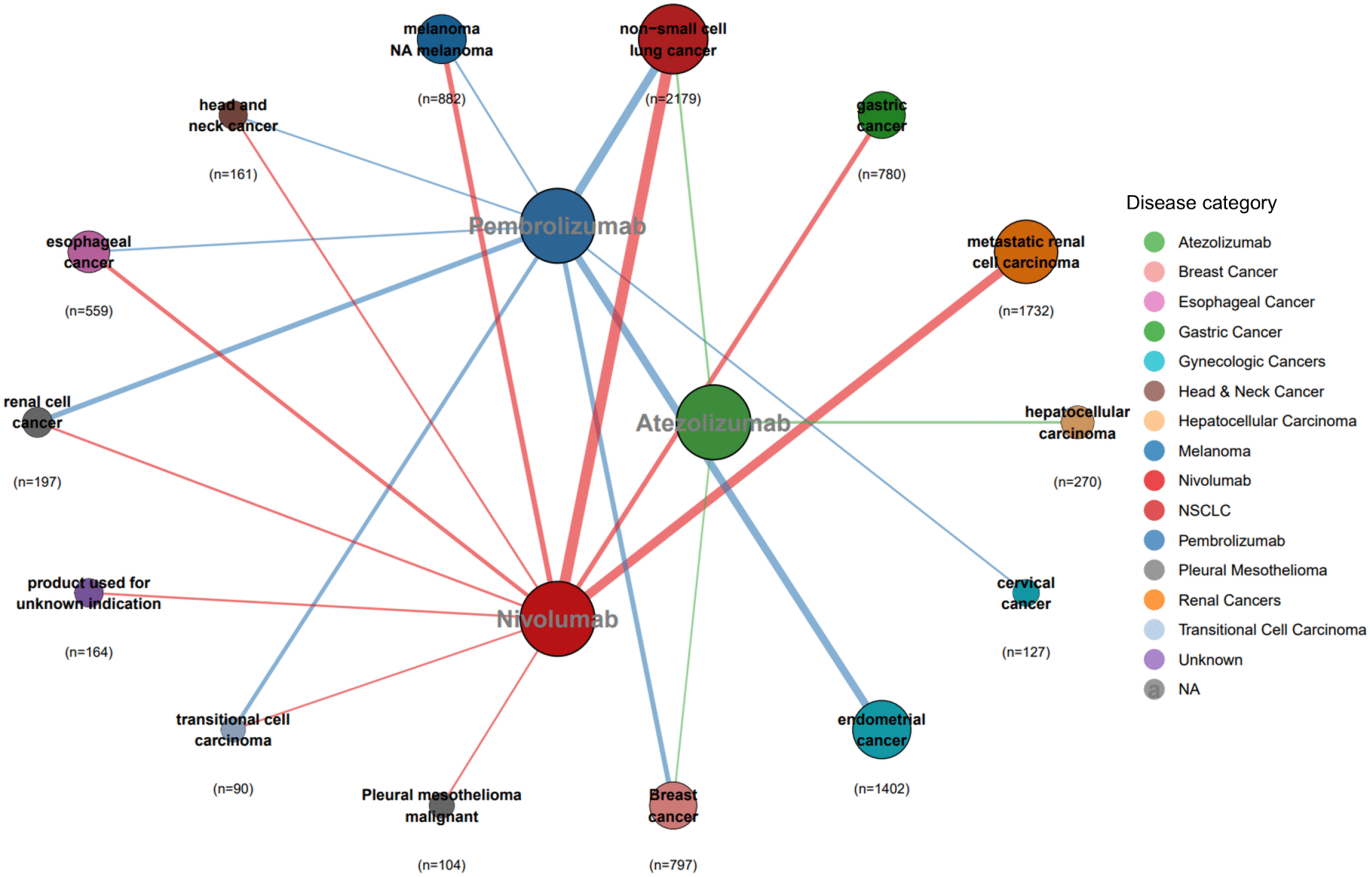
PD-1/PD-L1 Inhibitors Indications -- JADER (n = 9,444).

As depicted in Fig 5, JADER data indicate that nivolumab is indicated for non-small cell lung cancer, metastatic renal cell carcinoma, melanoma, gastric cancer, and esophageal cancer, among others. The indications for pembrolizumab include non-small cell lung cancer, endometrial carcinoma, renal cell carcinoma, breast cancer, and urothelial carcinoma, whereas atezolizumab is used primarily for hepatocellular carcinoma, non-small cell lung cancer, and breast cancer.

A cross-chart comparison reveals notable discrepancies in off-label uses beyond shared indications. In FAERS, nivolumab is used for clear cell renal cell carcinoma and Hodgkin’s lymphoma, whereas JADER documents its application in urothelial carcinoma and malignant pleural mesothelioma. Pembrolizumab shows distinct patterns: FAERS includes squamous cell lung carcinoma; however, JADER adds head and neck/cervical cancers to its indication list. Atezolizumab’s FAERS profile uniquely includes small cell lung cancer.

Furthermore, comparisons with FDA-approved drug inserts revealed supra-indicated uses. In the FAERS, 56 cases of pembrolizumab were reported for treating squamous cell lung carcinoma, and 87 cases of atezolizumab were documented for triple-negative breast cancer. Moreover, the JADER reported 67 cases in which atezolizumab was used for breast cancer treatment.

### Age‒sex comparison of patients with endocrine irAEs

**Fig 6:**
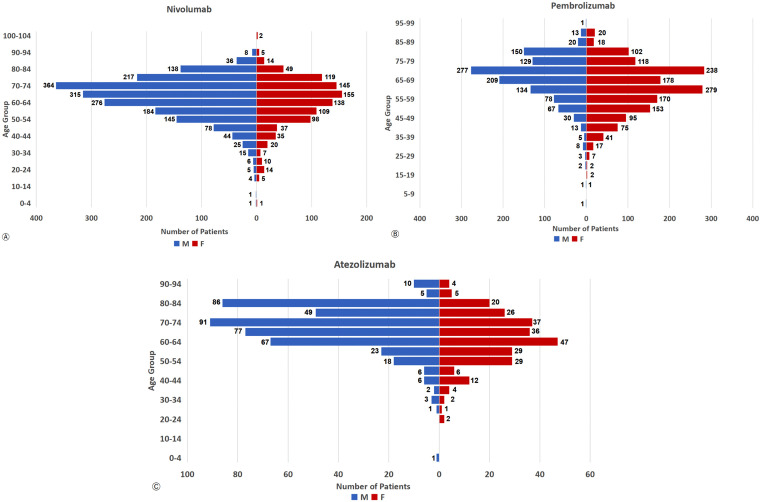
Population pyramid for endocrine riAEs -- FAERS (n_Nivo_ = 2,825, n_Pemb_ = 2,657, n_Atez_ = 705).

**Fig 7:**
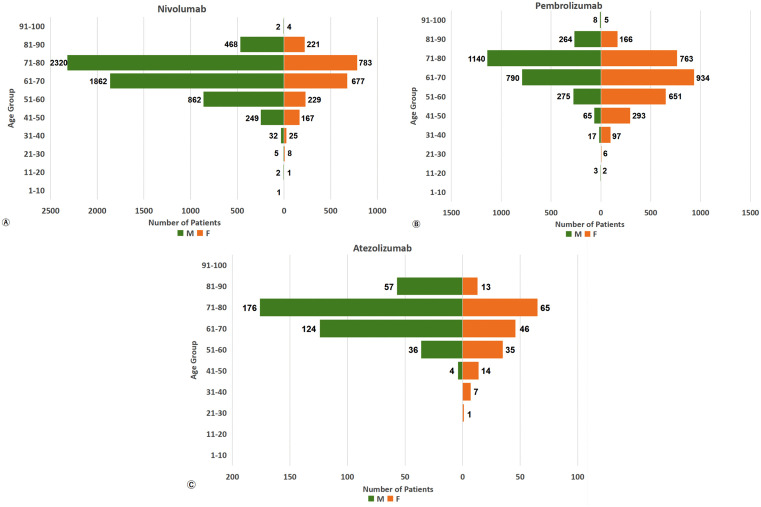
Endocrine riAEs population demographic pyramid -- JADER (n_Nivo_ = 7,918, n_Pemb_ = 5,479, n_Atez_ = 578).

The age and sex data of patients with nivolumab-, pembrolizumab-, and atezolizumab-related endocrine irAEs from the FAERS and JADER databases were analyzed and presented as population pyramids, as depicted in Figs 6 and 7, respectively.

In the FAERS, the reported cases of nivolumab-associated endocrine irAEs predominantly involved males, mainly those in the middle-aged and older age groups (50–84 years). However, for pembrolizumab, the majority of reported cases were females within the 50–84-year-old range. Similarly, atezolizumab-related irAEs reports were mainly from males, with the largest proportion falling into the 60–84-year-old age group.

The age and sex distributions of the endocrine irAEs populations in the JADER were approximately consistent with those in the FAERS.

### Distribution of outcomes for endocrine irAEs

**Fig 8:**
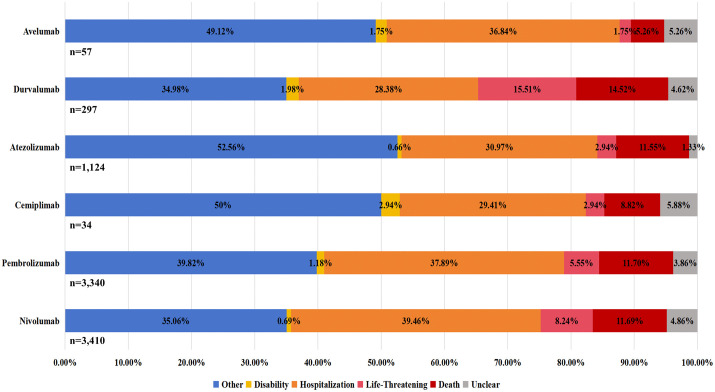
Distribution of outcomes for endocrine irAEs—FAERS.

**Fig 9:**
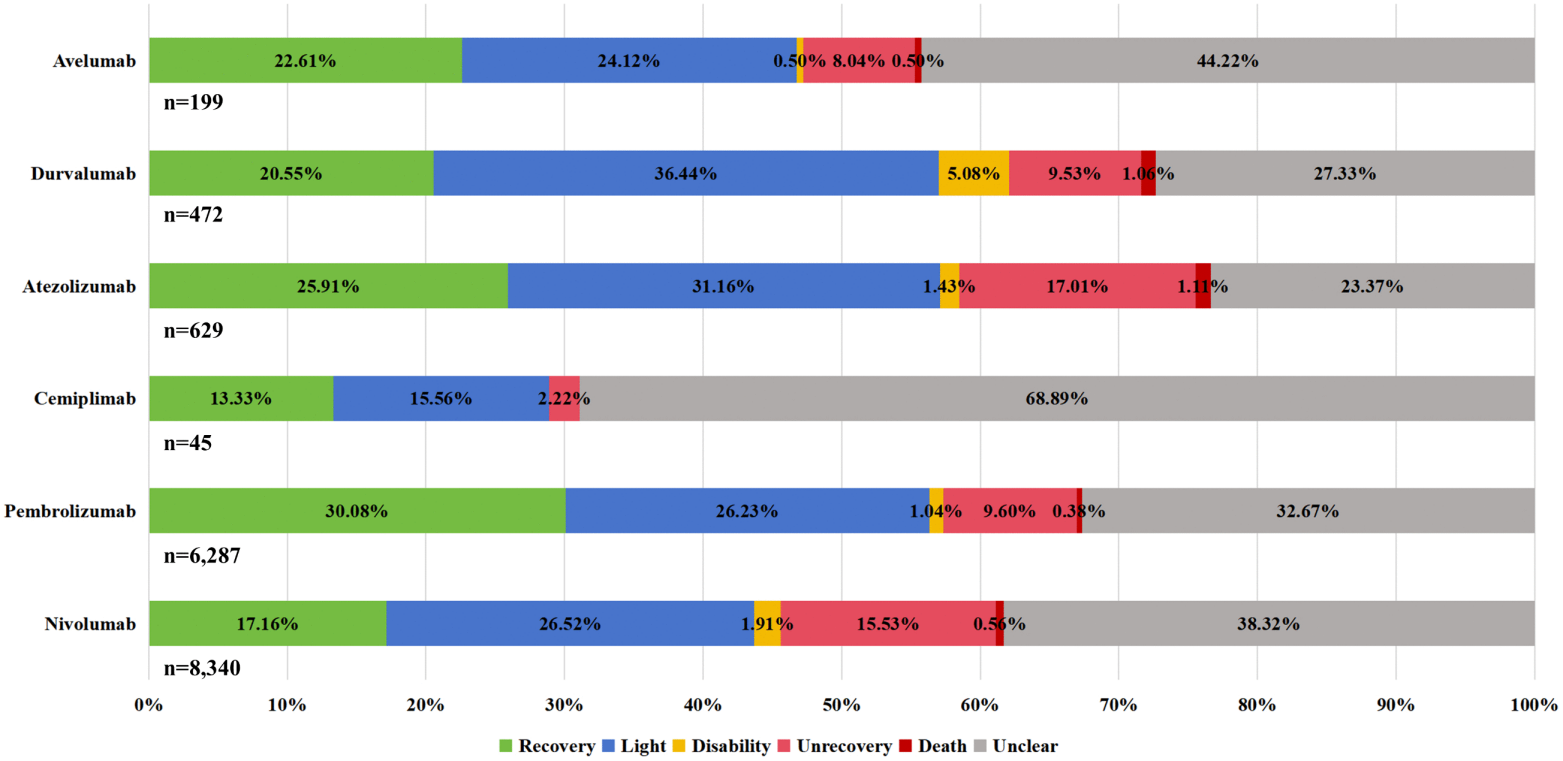
Distribution of outcomes for endocrine irAEs–JADER.

Percentage bar charts depicting the outcomes of endocrine irAEs from FAERS and JADER are presented in Figs 8 and 9, respectively. After excluding cases with unknown serious outcomes, analysis of FAERS data showed prolonged hospitalization (28.38%−39.46%) as the most common outcome of endocrine irAEs, followed by death (5.26%−14.52%). The proportion of fatal outcomes varied by drug: nivolumab (410/3,410, 12.02%), pembrolizumab (440/3,340, 13.17%), atezolizumab (128/1,124, 11.39%), and durvalumab (34/297, 11.45%) all exhibited mortality rates exceeding 11%. In contrast, JADER data revealed that the majority of patients had unknown outcomes. However, the death rate was relatively low, ranging from 0.38% to 1.11%, with specific proportions as follows: nivolumab (47/8,340, 0.56%), pembrolizumab (24/6,287, 0.38%), cemiplimab (1/45, 2.22%), atezolizumab (7/629, 1.11%), durvalumab (5/472, 1.06%), and avelumab (1/199, 0.50%). Therefore, a significant disparity in reported mortality was observed between the two databases, with FAERS exhibiting markedly higher fatal outcome proportions across all corresponding drugs.

### Time-to-onset analysis

Cumulative incidence curves were generated to analyze the time-to-onset of endocrine irAEs following treatment with five different immune checkpoint inhibitors (ICIs), in JADER. The majority of endocrine adverse events exhibited a rapid increase in cumulative incidence within the first 3 months of therapy.

**Fig 10:**
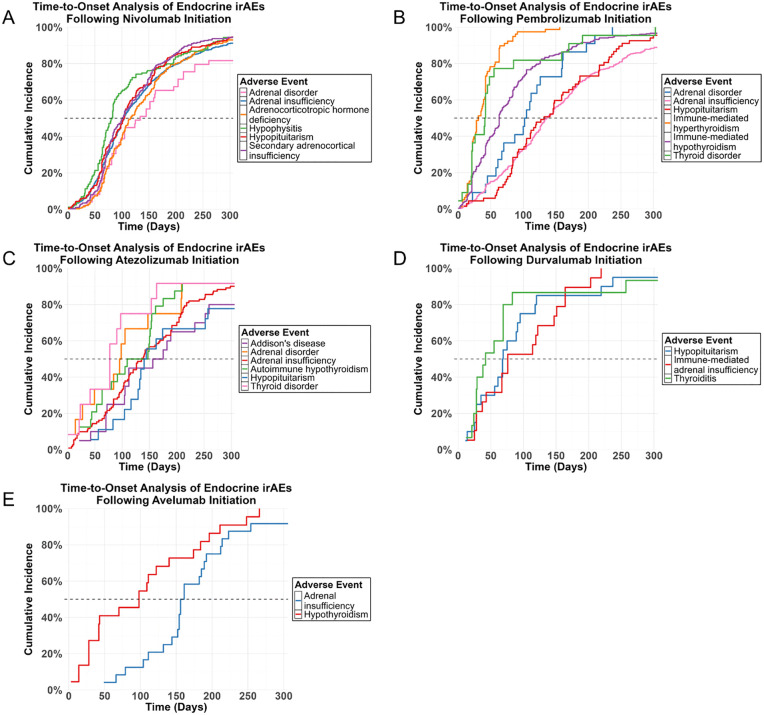
Comparative analysis of time-to-onset profiles for endocrine irAEs across five PD-1/PD-L1 inhibitors—JADER.

For Nivolumab (Fig 10A), hypophysitis occurred most rapidly, reaching a cumulative incidence of approximately 80% by day 100. In contrast, secondary adrenocortical insufficiency showed a slower progression. Regarding Pembrolizumab (Fig 10B), thyroid-related events dominated, with hyperthyroidism and immune-mediated hyperthyroidism peaking early (over 60% within 50 days). Atezolizumab (Fig 10C) demonstrated significant thyroid involvement, characterized by autoimmune hypothyroidism occurring within 100 days. Durvalumab (Fig 10D) similarly triggered rapid thyroid dysfunction, whereas Avelumab (Fig 10E) was associated with a delayed onset of adrenal insufficiency compared to other agents.

**Fig 11:**
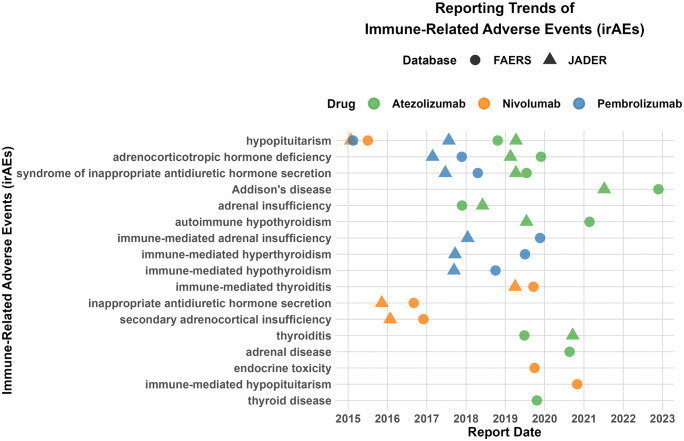
Temporal comparison of first reported irAEs for PD-1/PD-L1 inhibitors in FAERS and JADER databases.

As shown in Fig 11, systematic differences exist in the initial adverse event reporting timelines between the FAERS and JADER databases for the same drugs. For Pembrolizumab, initial reports in JADER were concentrated in 2015–2016, while in FAERS they primarily appeared during 2018–2020. Atezolizumab showed more initial reports in JADER earlier (around 2017), whereas significant reporting in FAERS occurred after 2019. Nivolumab’s initial reporting peaked in JADER during 2016–2018, compared to around 2020 in FAERS. Overall, JADER demonstrated earlier reporting, while FAERS reports were more concentrated in later periods, reflecting differences in reporting timeliness and regional drug use.

## Discussion

This study systematically analyzed the real-world safety profile of PD-1/PD-L1 inhibitors via data from the FAERS and JADER pharmacovigilance databases, marking the first systematic cross-database validation of endocrine irAEs induced by these inhibitors. By comparing FAERS and JADER, we assessed the consistency of signaling between regulatory systems and regional populations. In particular, the inclusion of JADER data provided novel safety insights specific to Asian populations, thereby contributing to global pharmacovigilance efforts and highlighting regional disparities in irAEs reporting.

Although ICIs have demonstrated remarkable efficacy in treating various cancers, the diagnosis and management of their associated endocrine irAEs remain significant challenges for healthcare providers. This study aimed to systematically analyze endocrine irAEs associated with FDA-approved and marketed PD-1/PD-L1 inhibitors in the FAERS and JADER databases. By examining case report frequencies, correlations with monoclonal antibodies, distributions of reporting countries, categories of endocrine irAEs, indications for PD-1/PD-L1 inhibitors, age‒sex demographics, and patient outcomes, the following key findings were obtained:

### Basic characteristics of the population of endocrine irAEs associated with PD-1/PD-L1 inhibitors

First, our results show that the reporting volumes for nivolumab and pembrolizumab in the JADER database far exceed those in FAERS. The most direct explanation for this phenomenon is the localized nature of JADER. As a database primarily comprising Japanese patient data, JADER more comprehensively captures real-world safety information of PD-1/PD-L1 inhibitors widely used in Japan. This suggests that relying on a single database (e.g., FAERS alone) for evaluating global drug safety profiles may lead to underestimation of region-specific safety signals, particularly in regions like Asia. Therefore, multinational, multi-database comparative studies are essential for constructing a comprehensive drug safety profile. This trend might be attributed to a lower awareness of endocrine irAEs in other countries or differences in the size and characteristics of populations using these drugs.

In both the FAERS and the JADER, the age‒sex compositions of the population showed notable similarities. Specifically, among patients with endocrine irAEs, males accounted for a greater proportion than females did for both nivolumab and atezolizumab, with middle-aged and elderly individuals aged 50–84 years being the predominant group. In contrast, Pembrolizumab targets cancers more common in women, such as endometrial and cervical cancer. Consequently, reports of its endocrine irAEs were slightly higher in females, while maintaining highest frequency in the 50−84 age range. Overall, male patients presented a greater prevalence of endocrine irAEs associated with PD-1/PD-L1 inhibitors. This observed higher risk in males contrasts with the general pattern of females being more prone to stronger immune responses and autoimmune diseases [17]. Several factors may explain this discrepancy. First, as FAERS and JADER are spontaneous reporting databases with diverse data sources, the accuracy of reported information cannot be fully guaranteed. Specifically, our analysis revealed instances of missing gender data, and misreporting remains a potential confounding factor. These findings underscore the need for heightened awareness of treatment-related adverse events among middle-aged and elderly males aged 50 years and older. Therefore, enhancing baseline and periodic monitoring for endocrine system—such as regular thyroid and adrenal function tests—in this specific population could be warranted to enable earlier detection and intervention.

### Correlations of endocrine irAEs with PD-1/PD-L1 inhibitors

This study compared the signaling of endocrine irAEs associated with PD-1/PD-L1 inhibitors in the FAERS and JADER databases, identifying remarkable disparities in signal intensity between the two datasets. These discrepancies not only mirror differences in reporting mechanisms and data origins, but also imply that Asian patients might manifest distinct patterns of irAEs occurrence. Consequently, further research is warranted to elucidate these observations and inform clinical decision-making.

Cross-database comparison revealed striking disparities in irAEs correlations. In the JADER, the correlation between atezolizumab and autoimmune hypothyroidism increased to 266.7, significantly surpassing that in the FAERS. Similarly, cemiplimab demonstrated a notable association with hypothyroidism (ROR = 64.9) in JADER, whereas no such link was detected in FAERS, suggesting a potentially higher prevalence of these irAEs among Asian populations. In contrast, for most irAEs, including thyroiditis associated with nivolumab and secondary adrenocortical insufficiency related to cemiplimab, FAERS reported stronger correlations, likely due to signal overestimation from lay reporting.

Differences in healthcare systems, reporting practices, and population genetics can profoundly impact the interpretation of pharmacovigilance data, highlighting the multifactorial nature of irAEs signal discrepancies between the two databases [18]. In particular, JADER’s data exclusively stem from healthcare organizations, ensuring higher data integrity while also biasing the reporting toward clinically significant irAEs. This characteristic may explain the heightened signals for specific irAEs. And the stronger correlation of pituitary irAEs in FAERS, along with the identification of endocrine irAEs not documented in drug labels, may be attributed to the FAERS’ open reporting system, which enables consumers to submit irAEs reports. This mechanism increases the likelihood of capturing subjectively perceived irAEs. Additionally, variability in routine screening practices across healthcare systems directly influences the reported incidence and signal strength of irAEs in different databases.

Furthermore, differences in regulatory priorities and clinical reporting standards further exacerbate the divergence in irAEs signal distributions between FAERS and JADER, highlighting the complex interplay of multiple factors in pharmacovigilance data interpretation.

Finally, population genetics offers a biological basis for irAE susceptibility. Differences between Asian-predominant JADER and diverse FAERS data may arise from population-specific variations in immune genes, such as HLA alleles, which directly affect individual risk. For example, hypothyroidism has been reported to be more common in certain Asian countries than in developed European nations [19].

These findings underscore that irAEs risk is not uniform and is influenced by regional and demographic factors. For clinicians, this implies that monitoring protocols should be tailored to the specific PD-1/PD-L1 inhibitor used and the patient’s ethnic background.

Based on disproportionality analysis of the FAERS database, this study detected potential safety signals for SIADH associated with nivolumab, pembrolizumab, and atezolizumab. It is important to emphasize that signal detection indicates statistical association, not causation; these findings should be considered hypothesis-generating and guide further investigation. Biologically, endocrine irAEs often involve the pituitary gland, and antidiuretic hormone secretion is regulated by the hypothalamic-pituitary axis [20]. Therefore, we might infer that if PD-1/PD-L1 inhibitors disrupt this axis, SIADH could manifest as a clinical outcome. These results suggest a hypothesis requiring validation: some observed adverse events may be components of a broader immune-related endocrine syndrome.

To enhance patient safety, we recommend that clinicians maintain a high suspicion for SIADH in patients presenting with hyponatremia or nonspecific neurological symptoms. Incorporating routine serum sodium monitoring can facilitate earlier diagnosis and management [20].

In summary, this study suggests that certain irAEs identified through pharmacovigilance may be secondary manifestations of other, initially unrecognized primary irAEs [21].

An analysis of irAEs with strong correlations in both the FAERS and JADER databases revealed that PD-1/PD-L1 inhibitors were predominantly associated with thyroid dysfunction, adrenal insufficiency, and pituitary inflammation. A detailed examination of these three irAEs led to the following key findings:

### Thyroid dysfunction

In this study, thyroid dysfunction emerged as the most frequently reported endocrine irAEs, which aligns closely with clinical experience. This finding underscores the prevalence of thyroid disorders as the most common adverse endocrine reactions during PD-1/PD-L1 inhibitor therapy [22]. Among these, hypothyroidism accounted for the greatest number of reports, followed by hyperthyroidism. Given that the thyroid gland appears to be a preferential target for drugs that modulate immune responses in noncancer diseases, these results suggest that the thyroid is highly susceptible to immunomodulatory therapies [23].

Although several studies have shown a correlation between thyroid dysfunction and enhanced overall survival in cancer patients, the underlying mechanism remains elusive [24]. The high rate of thyroid disorders during immunotherapy likely stems from two factors: widespread genetic susceptibility to thyroid autoimmunity and the common presence of antithyroid antibodies in the population [25]. In addition, no specific risk factors, including age or sex, have been identified as contributing to individual susceptibility to thyroid disease within the context of endocrine irAEs. Hypothyroidism typically results from destructive thyroiditis. However, the prevalence of thyroiditis might be underestimated, as most clinical trials have treated hypothyroidism and hyperthyroidism as distinct conditions rather than sequential stages of a single thyroiditis case [26,27].

Studies have demonstrated that more than 50% of patients receiving PD-1/PD-L1 inhibitors initially develop hypothyroidism. Given that most of these patients remain asymptomatic, this condition often progresses to hyperthyroidism at a later stage [28].

Given that the majority of patients receiving PD-1/PD-L1 inhibitor therapy exhibit asymptomatic thyroid dysfunction, regular screening by healthcare professionals is imperative. In accordance with the clinical practice guidelines from the ESMO, ASCO, NCCN, and SITC, thyroid function tests should be conducted every 4–6 weeks. This protocol enables the early detection of potential progression to the hypothyroid stage [4,29].

### Pituitary gland inflammation

Even if most patients with pituitary irAEs present with pronounced symptoms, differentiating these from other acute diseases or potential malignancies remains a diagnostic challenge for clinicians [4]. Pituitary irAEs can disrupt multiple endocrine axes, resulting in central hypothyroidism, secondary adrenal insufficiency, hypogonadotropic hypogonadism, or, less commonly, central enuresis [7]. Like thyroid irAEs, ICI-related therapy should be suspended until severe symptoms abate and appropriate hormone replacement therapy is initiated upon the onset of pituitary inflammation. Immunotherapy may then be resumed once the condition resolves [27]. Specifically, the incidence of pituitary inflammation is lower (0.8%) with the combination of LAG-3 and PD-1 inhibitors than with PD-1 monotherapy (2.5%) [30]. While specific risk factors for pituitary inflammation have yet to be definitively identified, small-scale studies suggest that age and male sex may be potential contributors, as males and older patients are at greater risk [31].

### Adrenal insufficiency

Adrenal insufficiency is characterized by insufficient production of cortisol in the adrenal cortex [32]. However, clinical studies reporting primary adrenal insufficiency induced by ICIs are relatively rare [7]. This scarcity can likely be ascribed to the concurrent use of glucocorticoids or the presence of secondary adrenal insufficiency, both of which might result in an underestimated prevalence [33]. Patients with ICI-induced adrenal insufficiency commonly exhibit symptoms such as fatigue, malaise, nausea, and hypokalemia. In severe instances, an adrenal crisis may ensue, which, if left untreated, can be life-threatening [34]. Reportedly, elderly individuals and men are at increased risk of developing ICI-related primary adrenal insufficiency. Moreover, this condition may contribute to poorer clinical outcomes among patients with low body weight [35].

### Indications for PD-1/PD-L1 inhibitors

The indicated uses of PD-1/PD-L1 inhibitors differ substantially between countries. These disparities likely reflect regional variations in treatment preferences or clinical guidelines. For example, nivolumab is indicated for Hodgkin’s lymphoma in FAERS but not in JADER. This distribution pattern aligns with known epidemiology: Hodgkin’s lymphoma incidence is higher in North America and Europe but significantly lower in Asian populations [36,37].

When each database is separately compared with the FDA-approved indications in PD-1/PD-L1 inhibitor drug inserts, the data highlight extensive off-label use of these medications. Clinical use of these drugs often expands ahead of formal label updates, suggesting that practice changes faster than official documentation. This scenario underscores the critical importance of safety monitoring, as off-label use may entail unforeseen safety risks, including potential drug misuse. Strengthened adverse event surveillance, such as through platforms such as FAERS and JADER, is indispensable for evaluating the safety of these usage patterns.

Additionally, off-label data may identify therapeutic areas worthy of further exploration. The use of pembrolizumab in lung squamous cell carcinoma and atezolizumab in triple-negative breast cancer warrants further clinical trials to confirm efficacy and safety.

### Endocrine irAEs outcomes associated with PD-1/PD-L1 inhibitors

A marked divergence in reported outcomes was observed between the FAERS and JADER databases. FAERS data indicated a considerable burden of serious sequelae from endocrine irAEs, including prolonged hospitalization and notably higher mortality. Conversely, JADER data were characterized by predominantly non-fatal outcomes and significantly lower reported mortality [38].

This pronounced difference likely reflects fundamental divergences in pharmacovigilance systems rather than true variation in drug safety, as FAERS’s broader reporting scope contrasts with JADER’s stringent medical validation. These findings underscore the need for vigilant monitoring of patients receiving PD-1/PD-L1 inhibitors, while highlighting how reporting system characteristics shape pharmacovigilance data interpretation.

### Limitations

This study has several limitations inherent to its use of spontaneous reporting systems. While the FAERS and JADER databases are invaluable for pharmacovigilance, their reliance on voluntary reports leads to challenges such as under-reporting, missing data, reporting delays, and potential information bias. A fundamental constraint is the general lack of detailed clinical context, which significantly impedes a nuanced interpretation of the detected signals. Crucial patient-specific information—such as detailed medical history, underlying comorbidities, concurrent medications, and objective laboratory or imaging data—is frequently absent. This gap makes it impossible to control for key confounding factors [39]. Similarly, without complete medication records, the influence of concomitant drugs on the reported endocrine irAEs cannot be ruled out. Consequently, the absence of this clinical granularity remains a major constraint that can lead to either overestimation or underestimation of true associations.

Furthermore, the disparity in data quality and sources between the two databases must be acknowledged. The FAERS database aggregates reports from consumers, clinicians, and manufacturers, which may include unverified or incomplete cases. In contrast, JADER primarily collects reports from healthcare organizations, thereby ensuring a generally higher standard of data validity and completeness. Despite our strategy of cross-validating signals between FAERS and JADER to enhance robustness, the findings from our disproportionality analysis must be interpreted as hypothesis-generating. Methods like the ROR are powerful for detecting statistical associations but cannot establish causality and carry an inherent risk of both false-positive and false-negative results.

A key limitation of this pharmacovigilance study is the inherent lack of denominator data, specifically the total number of patients exposed to each drug. Consequently, the reported ROR values must be interpreted as measures of relative proportion within the database, not as estimates of incidence or absolute risk in the treated population. Moreover, regional variations in prescription volumes, clinical practices, and reporting culture can significantly influence the strength of the observed signals, independent of the true underlying drug-event risk [40,41].

## Conclusion

In this study, the FAERS and JADER databases were employed to conduct an in-depth analysis of endocrine irAEs associated with PD-1/PD-L1 inhibitors. Several endocrine irAEs not documented in drug inserts, including inappropriate antidiuretic hormone secretion syndrome, urosepsis, mucous edema, and hypopituitarism, have been identified. Although these events are relatively rare, they necessitate close clinical monitoring, particularly during long-term therapy.

By leveraging dual database validation, this study offers a more comprehensive and regionally diverse safety evaluation of the endocrine effects of PD-1/PD-L1 inhibitors. These findings underscore the critical importance of focusing on the thyroid, adrenal, and pituitary glands, as well as the necessity of individualized patient monitoring when these drugs are administered. Clinicians should educate ICI recipients about potential toxicities and recognize the distinct endocrine risks of different regimens to facilitate timely management.

As proposed by Aung Naing et al. in a recent publication [42], strategies such as enhancing patient education, leveraging technology for early irAEs detection and timely intervention, and conducting further research to deepen our understanding of irAEs and develop personalized management approaches are essential for improving the overall management of irAEs.

## Supporting information

S1 Table Consolidated summary of disproportionality analysis for PD-1/PD-L1 inhibitors and endocrine irAEs from FAERS and JADER. Abbreviations: ROR: Reporting Odds Ratio. a: Number of reports containing both the target drug and the target adverse event. b: Number of reports containing the target drug but not the target adverse event. c: Number of reports not containing the target drug but containing the target adverse event. d: Number of reports containing neither the target drug nor the target adverse event. SIADH: Syndrome of Inappropriate Antidiuretic Hormone Secretion.
(DOCX)
