# Correction: Endocrine advantages of PD-1/PD-L1 therapy: Comparative analysis of FAERS-JADER

**DOI:** 10.1371/journal.pone.0344694

**Published:** 2026-03-10

**Authors:** Yuxuan Gao, Shiyao Jiang, Yu Cui, Yumeng Wang, Lili Yu

The Funding statement for this article is incomplete. The correct Funding statement is as follows: This work was supported by the Shandong Second Medical University(China), Postgraduate Research Innovation Fund Project [Project Number: 2023YJSCX023], Recipient: Yuxuan Gao; the Shandong Province Medical and Health Technology Project(China) [Project Number: 202412051156], Recipient: Lili Yu; and the Science and Technology Development Project for the Affiliated Hospital of Shandong Second Medical University(China) [Project Number: 2023FYZ015], Recipient: Lili Yu.
