# Supplementary material for: Endocrine advantages of PD-1/PD-L1 therapy: Comparative analysis of FAERS-JADER
Source: PLoS One. 2026 Jan 9;21(1):e0340794. doi: 10.1371/journal.pone.0340794 (PMC12788665; doi:10.1371/journal.pone.0340794)
Supplement: S1 Table — Abbreviations: ROR: Reporting Odds Ratio. a: Number of reports containing both the target drug and the target adverse event. b: Number of reports containing the target drug but not the target adverse event. c: Number of reports not containing the target drug but containing the target adverse event. d: Number of reports containing neither the target drug nor the target adverse event. SIADH: Syndrome of Inappropriate Antidiuretic Hormone Secretion. (DOCX) [file pone.0340794.s001.docx]

The key signals selected for detailed contingency table presentation in the supplementary materials were based on a combination of clinical relevance (e.g., seriousness, novelty), statistical strength (e.g., ROR and lower 95% confidence interval), and consistency across the FAERS and JADER databases.

S1 Table : Consolidated Summary of Disproportionality Analysis for PD-1/PD-L1 Inhibitors and Endocrine irAEs from FAERS and JADER

| # | Database | Drug | Targeted Adverse Event | a | b | c | d | ROR |
| --- | --- | --- | --- | --- | --- | --- | --- | --- |
| 1 | FAERS | Nivolumab | Endocrine toxicity | 25 | 35,750 | 34 | 9,602,381 | 197.499 |
| 2 | FAERS | Nivolumab | Immune-mediated hypophysitis | 77 | 35,698 | 137 | 9,602,278 | 151.182 |
| 3 | FAERS | Nivolumab | Immune-mediated thyroiditis | 75 | 35,700 | 161 | 9,602,254 | 125.297 |
| 4 | FAERS | Nivolumab | Adrenocorticotropic hormone deficiency | 125 | 35,650 | 350 | 9,602,065 | 96.194 |
| 5 | FAERS | Nivolumab | Diabetes insipidus | 20 | 35,755 | 936 | 9,601,479 | 5.738 |
| 6 | FAERS | Nivolumab | SIADH | 46 | 35,729 | 3,832 | 9,598,583 | 3.225 |
| 7 | FAERS | Pembrolizumab | Immune-mediated hypothyroidism | 273 | 30,148 | 242 | 9,607,527 | 359.501 |
| 8 | FAERS | Pembrolizumab | Immune-mediated hyperthyroidism | 61 | 30,360 | 59 | 9,607,710 | 327.187 |
| 9 | FAERS | Pembrolizumab | Immune-mediated adrenal insufficiency | 129 | 30,292 | 161 | 9,607,608 | 254.127 |
| 10 | FAERS | Pembrolizumab | Adrenocorticotropic hormone deficiency | 166 | 30,255 | 309 | 9,607,460 | 170.593 |
| 11 | FAERS | Pembrolizumab | Diabetes insipidus | 11 | 30,410 | 945 | 9,606,824 | 3.677 |
| 12 | FAERS | Pembrolizumab | SIADH | 42 | 30,379 | 3,836 | 9,603,933 | 3.461 |
| 13 | FAERS | Cemiplimab | Secondary adrenocortical insufficiency | 4 | 1,040 | 784 | 9,636,362 | 47.274 |
| 14 | FAERS | Cemiplimab | Hypophysitis | 5 | 1,039 | 1,002 | 9,363,144 | 46.280 |
| 15 | FAERS | Cemiplimab | Autoimmune thyroiditis | 3 | 1,041 | 1,357 | 9,635,789 | 20.463 |
| 16 | FAERS | Atezolizumab | Hypopituitarism | 51 | 13,860 | 820 | 9,623,459 | 43.184 |
| 17 | FAERS | Atezolizumab | Adrenocorticotropic hormone deficiency | 27 | 13,884 | 448 | 9,623,831 | 41.775 |
| 18 | FAERS | Atezolizumab | Hypophysitis | 55 | 13,856 | 952 | 9,623,327 | 40.125 |
| 19 | FAERS | Atezolizumab | Adrenal insufficiency | 251 | 13,660 | 4,716 | 9,619,563 | 37.480 |
| 20 | FAERS | Atezolizumab | Endocrine toxicity | 8 | 13,903 | 51 | 9,624,228 | 108.587 |
| 21 | FAERS | Atezolizumab | SIADH | 26 | 13,885 | 3,852 | 9,620,427 | 4.677 |
| 22 | FAERS | Avelumab | Adrenal disorder | 5 | 1,286 | 760 | 9,636,139 | 49.297 |
| 23 | FAERS | Avelumab | Thyroiditis | 8 | 1,283 | 1,436 | 9,635,463 | 41.839 |
| 24 | FAERS | Avelumab | Thyroid disorder | 10 | 1,281 | 5,984 | 9,630,915 | 12.564 |
| 25 | FAERS | Durvalumab | Adrenocorticotropic hormone deficiency | 13 | 6,141 | 462 | 9,631,574 | 44.133 |
| 26 | FAERS | Durvalumab | Immune-mediated hypothyroidism | 10 | 6,144 | 505 | 9,631,531 | 31.042 |
| 27 | FAERS | Durvalumab | Immune-mediated hypophysitis | 4 | 6,150 | 210 | 9,631,826 | 29.831 |
| 28 | FAERS | Durvalumab | Adrenal disorder | 13 | 6,141 | 752 | 9,631,284 | 27.113 |
| 29 | JADER | Nivolumab | Hypophysitis | 281 | 49,210 | 116 | 1,508,644 | 74.250 |
| 30 | JADER | Nivolumab | Pituitary enlargement | 14 | 49,477 | 6 | 1,508,754 | 71.150 |
| 31 | JADER | Nivolumab | Secondary adrenocortical insufficiency | 464 | 49,027 | 230 | 1,508,530 | 62.070 |
| 32 | JADER | Nivolumab | Adrenocorticotropic hormone deficiency | 491 | 49,000 | 263 | 1,508,497 | 57.470 |
| 33 | JADER | Nivolumab | Immune-mediated hypophysitis | 102 | 49,389 | 56 | 1,508,704 | 55.460 |
| 34 | JADER | Pembrolizumab | Immune-mediated hypothyroidism | 1,045 | 36,194 | 249 | 1,520,763 | 176.340 |
| 35 | JADER | Pembrolizumab | Immune-mediated hyperthyroidism | 217 | 37,022 | 53 | 1,520,959 | 168.130 |
| 36 | JADER | Pembrolizumab | Immune-mediated adrenal insufficiency | 523 | 36,716 | 381 | 1,520,631 | 56.850 |
| 37 | JADER | Pembrolizumab | Immune-mediated thyroiditis | 71 | 37,168 | 69 | 1,520,943 | 42.110 |
| 38 | JADER | Pembrolizumab | Hypothalamo-pituitary disorder | 108 | 37,131 | 147 | 1,520,865 | 30.090 |
| 39 | JADER | Pembrolizumab | Hypopituitarism | 147 | 37,092 | 650 | 1,520,362 | 9.270 |
| 40 | JADER | Cemiplimab | Hypothyroidism | 32 | 139 | 5,511 | 1,552,569 | 64.860 |
| 41 | JADER | Cemiplimab | Hyperthyroidism | 8 | 163 | 2,291 | 1,555,789 | 33.330 |
| 42 | JADER | Cemiplimab | Adrenal insufficiency | 5 | 166 | 4,423 | 1,553,657 | 10.580 |
| 43 | JADER | Atezolizumab | Autoimmune hypothyroidism | 26 | 12,558 | 12 | 1,525,655 | 266.680 |
| 44 | JADER | Atezolizumab | Addison's disease | 23 | 12,561 | 22 | 1,545,645 | 128.640 |
| 45 | JADER | Atezolizumab | Adrenal disorder | 20 | 12,564 | 168 | 1,545,499 | 14.640 |
| 46 | JADER | Atezolizumab | Thyroid disorder | 19 | 12,565 | 218 | 1,545,449 | 10.720 |
| 47 | JADER | Avelumab | Hypothyroidism | 92 | 1,450 | 5,451 | 1,551,258 | 18.060 |
| 48 | JADER | Avelumab | Adrenal insufficiency | 57 | 1,485 | 4,371 | 1,552,338 | 13.630 |
| 49 | JADER | Avelumab | Hyperthyroidism | 20 | 1,522 | 2,279 | 1,554,430 | 8.960 |
| 50 | JADER | Durvalumab | Hypophysitis | 32 | 9,766 | 365 | 1,548,088 | 13.900 |
| 51 | JADER | Durvalumab | Immune-mediated hypophysitis | 12 | 9,786 | 146 | 1,548,307 | 13.000 |
| 52 | JADER | Durvalumab | Adrenal disorder | 13 | 9,785 | 175 | 1,548,278 | 11.750 |
| 53 | JADER | Durvalumab | Hypopituitarism | 42 | 9,756 | 755 | 1,547,698 | 8.830 |
| 54 | JADER | Durvalumab | Immune-mediated adrenal insufficiency | 44 | 9,754 | 860 | 1,547,593 | 8.120 |

Abbreviations:

ROR: Reporting Odds Ratio

a: Number of reports containing both the target drug and the target adverse event.

b: Number of reports containing the target drug but not the target adverse event.

c: Number of reports not containing the target drug but containing the target adverse event.

d: Number of reports containing neither the target drug nor the target adverse event.

SIADH: Syndrome of Inappropriate Antidiuretic Hormone Secretion
